# Assessment of technical competence in anterolateral buttress plate fixation of tibial plateau fractures: a global Delphi consensus study

**DOI:** 10.1007/s00590-025-04654-1

**Published:** 2026-02-10

**Authors:** Maj Ósk Bruun Stangegård, Mads Emil Jacobsen, Leizl Joy Nayahangan, Monica Ghidinelli, Chitra Subramaniam, Kristoffer Borbjerg Hare, Lars Konge, Amandus Gustafsson

**Affiliations:** 1 Copenhagen Academy for Medical Education and Simulation, Rigshospitalet, Copenhagen, Capital Region, Denmark; 2 Department of Orthopedic Surgery, Center for Orthopedic Research and Innovation, Næstved, Slagelse, and Ringsted Hospitals, Region Zealand, Denmark, Denmark; 3 AO Foundation, AO Education Institute, Davos, Switzerland; 4 AO Foundation, AO North America, Wayne, Pennsylvania, USA; 5 Department of Orthopedic Surgery and Traumatology, Odense University Hospital, Region of Southern Denmark, Odense, Denmark; 6 Institute for Clinical Medicine, University of Copenhagen, Copenhagen, Denmark

**Keywords:** Assessment, Surgical skills, Competency-based medical education, Delphi, Tibial plateau fracture, Buttress plate

## Abstract

**Purpose:**

Tibial plateau fractures often require surgery, where surgeon competence is critical. Competency-based medical education requires valid objective assessment. The purpose of this study was to develop an expert consensus-based, procedure-specific assessment tool to evaluate technical competence in buttress plate fixation of tibial plateau fractures.

**Methods:**

International orthopaedic trauma educators participated in a four round online Delphi process to establish consensus on the content of the assessment tool. Round 1 identified potential parameters; Round 2 rated importance of each parameter; Round 3 addressed items outside this manuscript; Round 4 assigned weights (1–10).

**Results:**

Eighty-seven surgeons from 42 countries participated. Of 31 parameters identified, five were excluded in Round 2, leaving 26. Parameter weights ranged from 6.8 to 9.6, with anatomical fracture reduction rated highest.

**Conclusion:**

The resulting consensus-based assessment tool shows strong content validity and supports structured feedback for formative and summative use in surgical training.

## Introduction

Tibial plateau fractures (TPFs), which carry a high risk of complications, require meticulous management. The primary objectives in TPF surgery are anatomical articular reduction, stable fixation, and restoration of limb alignment [1]. Although the optimal treatment remains debated, open reduction and internal fixation (ORIF) with an anterolateral buttress plate is widely used for lateral TPFs without depression [2]. TPFs account for ~ 1% of fractures [3], limiting trainees’ hands-on experience. Additionally, operating room efficiency, duty-hour limits, and liability concerns all reduce exposure [4].

Historically, surgical training followed a time-based model [4], assuming skill correlated with total clinical time rather than exposure to specific procedures or objective assessments. This approach, however, may contribute to variable outcomes, even among experienced surgeons [5]. Residency programmes are increasingly adopting competency-based medical education (CBME), which emphasises knowledge, behaviours, and technical skills. Its success relies on objective, valid assessments that, beyond measuring proficiency, can provide structured feedback—a key driver of skill development [6]. The Objective Structured Assessment of Technical Skills (OSATS) is widely used but has limited validity in orthopaedic procedures dependent on biomechanical principles such as fracture reduction, implant positioning, and load-sharing construct design [7]. These factors are often underrepresented in generic rating scales, underscoring the need for assessment tools tailored to osteosynthesis procedures.

A general needs assessment identified buttress plating among the most important osteosynthesis techniques for inclusion in a simulation-based training (SBT) curriculum [8]. The need for structured assessment is reinforced by the designation of ORIF of proximal tibial fractures as a level 4 milestone in the ACGME Supplemental Guide for Orthopaedic Trauma Surgery [9] and by Kellam et al., who identified it as a core competency for general orthopaedic surgeons in the US [10]. In this context, an alternative feedback approach is needed – one that both assesses performance and supports skill development. To address this gap, we collaborated with an international panel of orthopaedic trauma experts to generate a list of essential assessment parameters forming a comprehensive procedure-specific assessment tool for evaluating technical competence in anterolateral buttress plate osteosynthesis for split fractures of the tibial plateau.

## Materials and methods

A global, modified Delphi process of four rounds was conducted. Ethical approval was sought from the Regional Ethics Committee of Region Zealand, Denmark, which determined the study was exempt (Journal number: EMN-2020-38838).

The Delphi method is widely accepted for establishing expert consensus and is suited to curriculum development and educational research. While experts agree on key issues, their perspectives are shaped by experience and training, leading to variation. The Delphi method builds consensus through diverse insights, iterative rounds, structured feedback, and anonymous participation. This approach reduces bias from dominant voices [11].

The four survey rounds were conducted from February to September 2021. Surveys were distributed to panellists via SurveyMonkey™, with response deadlines of 19–49 days and intervals between rounds of 14–49 days. The objective was to achieve expert consensus on components for developing assessment tools for seven distinct osteosyntheses. Results on distal radius locking plates and ulna compression plating have been published elsewhere. [12, 13] This report focuses on anterolateral buttress plate osteosynthesis for a simple lateral split fracture without depression (AO/OTA 41B1.1).

### Steering committee

The steering committee comprised seven members, including orthopaedic specialists and medical education researchers, who oversaw all aspects of study design, conduct, and analysis.

### Panellists

Potential panellists were identified through an AO Foundation email list of AO faculty trauma surgeons. Inclusion criteria required panellists to be: (1) faculty of the AO course “Basic Principles of Fracture Management” or (2) faculty of “Advanced Principles of Fracture Management” or (3) recommended by individuals meeting criteria (1) or (2). Exclusion criteria: (1) not an active trauma surgeon; (2) not supervising residents. Invitations were sent by email to 355 experts, providing a study overview and a link to the initial survey. Panellists who agreed to participate were invited to all subsequent rounds. Written consent was obtained from all participants.

## The Delphi rounds

### Round 1: generation of assessment parameters

Panellists were asked to respond to three questions:

(1) “Please list all the technical aspects (assessment parameters) that you find relevant to assess when determining how well a buttress plate osteosynthesis of a simple, split fracture of the lateral tibial plateau without depression of the articular surface (AO/OTA 41B1.1) is performed by a novice resident”; (2) “Based on your experience, please list the most common errors relating to residents’ technical performance of the osteosynthesis i.e., frequent/typical errors made by novice residents relating to osteosynthesis”; and (3) “Based on your experience, please list the most critical errors relating to residents’ technical performance of osteosynthesis i.e., severe errors that jeopardise the surgical result/patient outcome”.

All responses underwent a thorough content analysis before being consolidated and categorised into procedural steps by the steering committee. Exact duplicates were removed. Responses to questions 2 and 3 helped refine and clarify the assessment parameters identified in question 1. For example, the phrase “addressing associated ligamentous injury” was refined and combined with a phrase regarding range of motion into the assessment parameter “Examination of the knee for ligamentous injury and range of motion after fixation.” The parameters were grouped into four procedural steps: fracture preparation, hardware selection, fixation technique, and procedural documentation. The steering committee then evaluated the resulting assessment parameters based on the predetermined criteria outlined in Table 1.

**Table 1:** Prespecified evaluation criteria for assessment parameters in Delphi round 1

	Inclusion criteria
1	Assessment parameters evaluating technical performance/skills
2	Assessment parameters relating strictly to the performance of the osteosynthesis and fluoroscopic control
3	Assessment parameters that could be incorporated into automated assessment metrics on a virtual reality simulator

	Exclusion criteria
1	Assessment parameters relating to nontechnical skills (e.g., preoperative planning, communication, teamwork, etc.)
2	Assessment parameters relating to technical performance other than the osteosynthesis and fluoroscopic control (e.g., Exposure, soft tissue handling, closure, fluoroscopy of contralateral side for templating, etc.)
3	Assessment parameters relating to prespecified standardized settings of the procedure (e.g., patient position, osteosynthesis method, etc.)
4	Assessment parameters relating to time spent, as this parameter will be inherent in all assessments in the simulated setting.

## Round 2: rating of importance

Following review and consolidation, the revised list was returned to the panellists for rating. Each was asked to assess the importance of each parameter using a five-point Likert-like scale: (1) “not important”, (2) “somewhat important”, (3) “important”, (4) “very important”, and (5) “extremely important”. A predefined consensus criterion required an average score of 3 or higher for a parameter to advance.

### Round 3: establishment of optimal intervals and borderline errors

In this round, data were collected regarding a specific bone and fracture model linked to the ongoing development of a virtual reality simulator. As these data fall outside the scope of this report, they are not presented here.

## Round 4: weight assignment

In the final round, panellists were asked to assign a weight to each assessment parameter. Although all parameters had received an average rating of at least 3 “important” in Round 2, this step ensured that parameters did not necessarily carry equal weight in the final assessment score. Each parameter’s weight was calculated as the mean of the weights assigned by all panellists.

### Data analysis

**Fig. 1:**
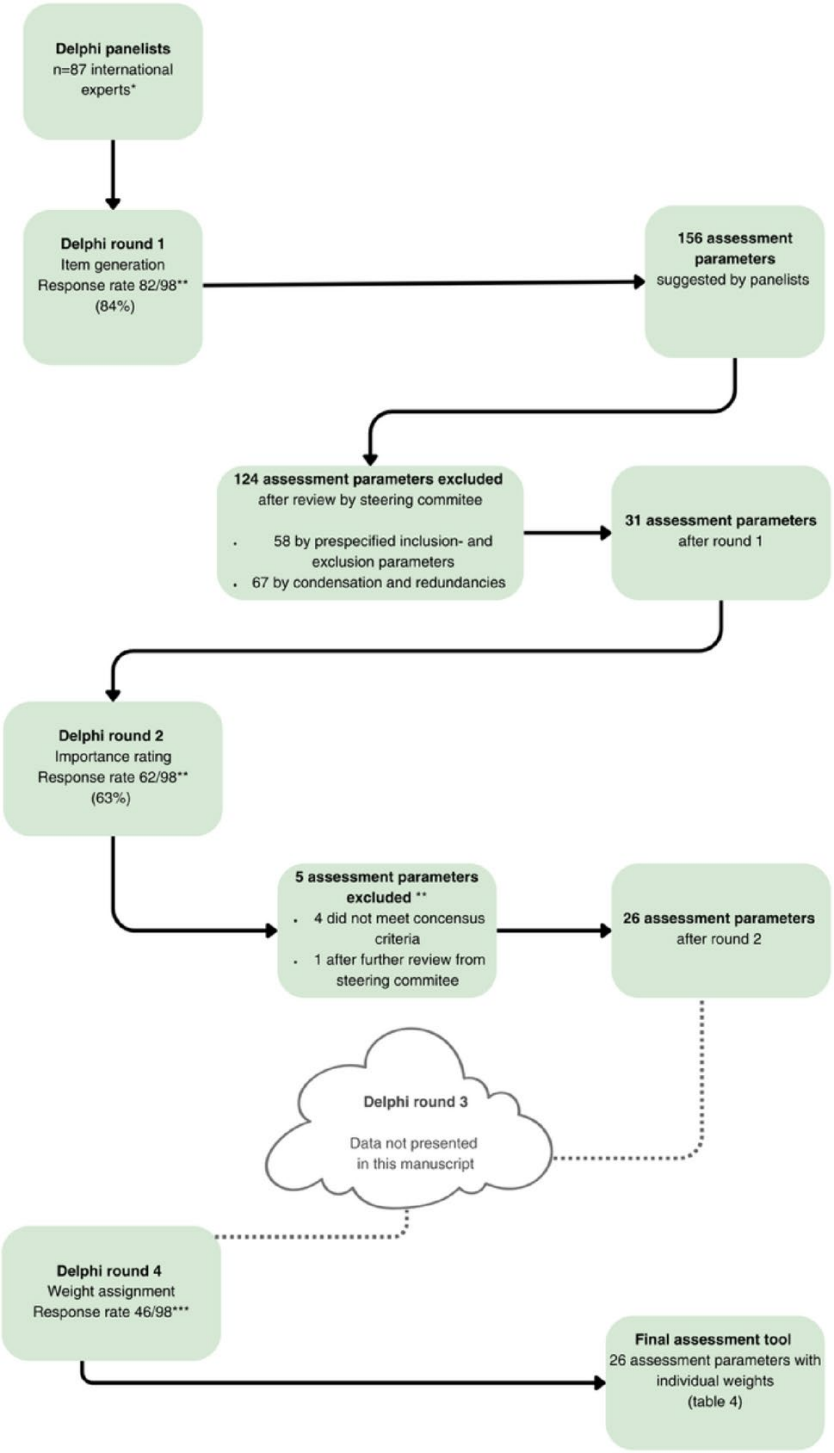
Flowchart of the Delphi process. * Number of participating panelistes. **See Table 3 for excluded parameters and the reasoning behind exclusion *** number of eligible surgeons who agreed to participate

Following Round 1, the steering committee performed a manual content analysis. For Rounds 2–4, descriptive statistics were applied. Correlations were calculated using Pearson’s r with 95% confidence intervals, and statistical significance was set at *p* < 0.05. Questionnaires with at least one response were classified as partial and, together with complete questionnaires, were included in response-rate calculations. All questionnaires, regardless of completeness, were included in the analysis shown in the flowchart  (Fig. 1).

## Results

**Fig. 2:**
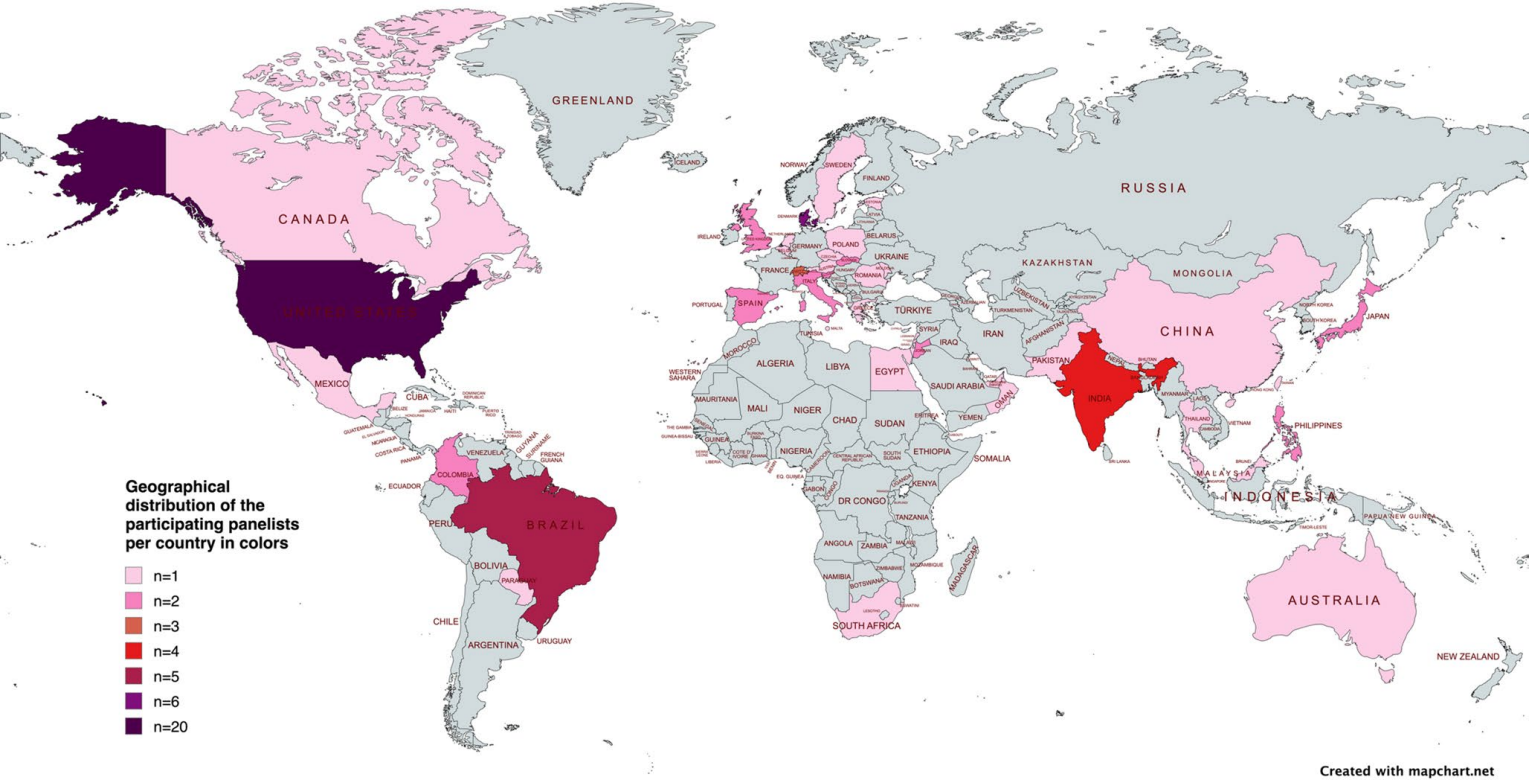
Geographical distribution of panellists per country *Created via mapchart.net*

Of the 355 experts invited via email, 119 accessed the survey link. Eleven did not provide any data, six declined to participate, and four did not meet the inclusion criteria. Ultimately, 98 individuals agreed to participate and provided the required personal information, resulting in a recruitment rate of 28%. Of these 98, 11 participants did not engage in any of the survey rounds and were consequently not considered panellists, reducing the final count of active panellists to 87. However, these 11 individuals were still included in response rate calculations. Details on the geographical distribution and characteristics of the panellists are presented in Table 2; Fig. 2.

**Table 2:** Characteristics of the panellists

	All panellists, *n* = 87	Panellists participating in all Delphi Rounds, *n* = 40
No. of years as an orthopaedic/trauma specialist, median (range):	17 (4–35)	17.5 (4–35)
Experience supervising surgical education of orthopaedic/trauma residents		
1-5y, *n*	1 (1,1%)	1 (2,5%)
6-10y, *n*	24 (27,6%)	10 (25%)
11-15y, *n*	17 (19,5%)	8 (20%)
More than 15y, *n*	45 (51,7%)	21(52,2%)
AO faculty, yes: no	86:1	40:0
AO, AO foundation y = years		

Table 2 is adapted with permission from: Jacobsen ME, Nayahangan LJ, Ghidinelli M, et al. (2023) Assessment of Technical Competence in Distal Radius Fracture Fixation by a Volar Locking Plate: a Global Delphi Consensus Study. *J Hand Surg Am*. 48(9):875–885 [12]

In Round 1, the response rate was 82 out of 98 (84%). No new assessment parameters emerged after reviewing two-thirds of the questionnaires. Panellists initially identified 156 parameters, reviewed by the steering committee. A total of 125 parameters were excluded: 58 based on prespecified criteria and 67 due to condensation and redundancy. After review, 31 remained.

In Round 2, the response rate was 62 out of 98 (63%). Five parameters were excluded – four for not meeting the consensus criteria (mean rating ≥ 3) and one upon further review by the steering committee, as it pertained to a standardised setting; ‘choice of plate thickness’ was excluded because it is predetermined by the selected implant type and does not constitute a trainee-level intraoperative decision. See Table 1 for exclusion criteria and Table 3 for examples of excluded parameters after Round 1 and all excluded parameters in Round 2.

The remaining parameters had a mean importance rating of 3.8 (SD 0.44), with individual means ranging from 3.0 to 4.8 (see Table 4).

**Table 3:** Assessment parameters excluded after Delphi rounds 1, 2 and 4

	Examples of assessment parameters excluded after round 1	
		*Reason*
1	Consider arthroscopy	Exclusion criterion 1
2	Neuromuscular assessment	Exclusion criterion 2
3	Position of the patient	Exclusion criterion 3
4	Size of plate	Redundant: Covered by assessment parameter 4: “Choice of plate length”

	Assessment parameters excluded in round 2	
		*Mean importance rating, SD*
1	Use of varus force to aid in the reduction (of anterolateral sagittal split)	2.96, 0.1
2	Use of a femoral distractor	2.58, 1.2
3	Number of reduction clamps	2.30, 0.95
4	Eccentric drilling in the hole for the apex screw	2.89, 1.26

	Assessment parameters excluded after further review by the steering committee after round 2	
		*Reason*
1	Choice of plate thickness	Exclusion criterion 3

**Fig. 3:**
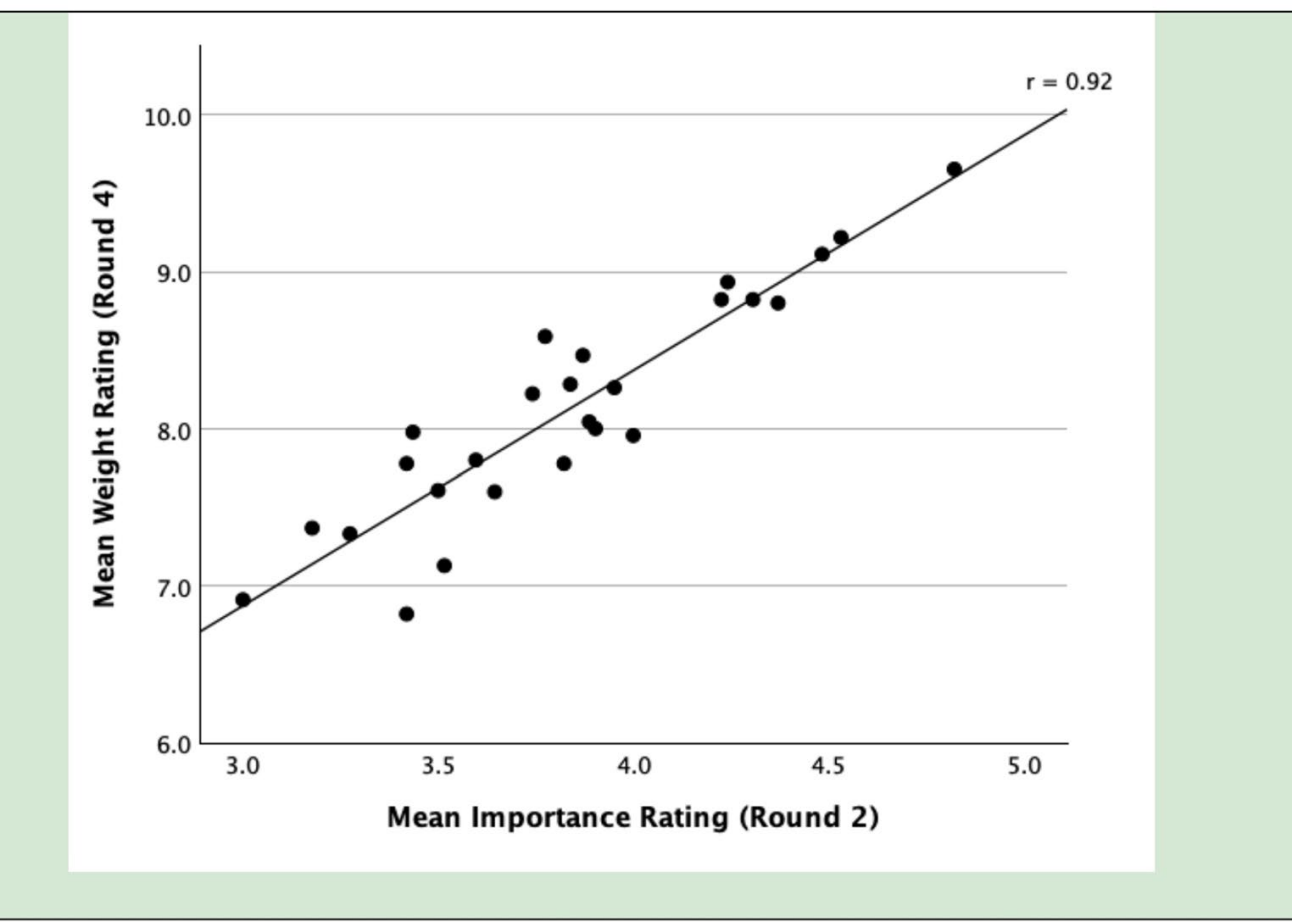
Scatterplot of mean importance ratings (Round 2) and mean weight ratings (Round 4) for the 26 assessment parameters, with fitted regression line. The annotated value (r) denotes Pearson’s correlation coefficient

In Round 4, the response rate was 46 out of 98 (47%). The 26 remaining parameters received a mean weight of 8.1 (SD 0.**73**), with individual means ranging from 6.8 to 9.7. A statistically significant, strong correlation was found between the mean importance rating in Round 2 and the mean weight in Round 4: Pearson’s *r* = 0.92 (95% CI: 0.83–0.96, *p* < 0.001), indicating that parameters rated as more important were also assigned higher weights. This association is illustrated in Fig. 3. The final list of parameters is presented in Table 4.

**Table 4:** Final list of assessment parameters for buttress plate osteosynthesis of a simple, split fracture of the lateral tibial plateau without depression of the articular surface (AO/OTA 41.B1) AO/OTA, AO Foundation/Orthopaedic trauma association fracture classification; Avg, average

Buttress plate osteosynthesis of a simple, split-fracture of the lateral tibial plateau without depression of the articular surface (AO/OTA 41B1.1)				
Step	Assessment parameter		Avg. Importance (Round 2)	Avg. Weight (Round 4)
Reduction	1	Fracture site preparation (removal of debris and hematoma, lavage of fracture and joint, and preparation of periosteum)	3.9	8.0
	2	Medial percutaneous incision for reduction clamp placement	3.2	7.4
	3	Placement of a reduction clamp to compress the fracture	3.8	8.6
	4	Anatomical fracture reduction	4.8	9.7
	5	Direct visualization of articular surface reduction (by arthrotomy)	3.5	7.6
	6	Use of the plate as a reduction tool	3.0	6.9
	7	Confirmation of fracture reduction using fluoroscopy prior to final fixation	4.5	9.2
Choice of hardware	8	Choice of plate length	3.5	7.1
	9	Choice of drill sizes	3.8	8.3
	10	Choice of screw types (cortical/cancellous, fully/partially threaded)	4.0	8.0
	11	Choice of screw diameter	3.8	7.8
Placement of plate	12	Contouring of the plate	3.3	7.3
	13	Distance from top of the plate to the joint line	4.0	8.3
	14	Placement of the plate relative to the direction of the shearing force (i.e., combination of the alignment (rotation) of the plate relative to the axis of tibia and the anterior-posterior translation of the plate in the sagittal view)	4.2	8.8
Placement of screws	15	Sequence of screw placement	3.9	8.0
	16	Number of screws below the fracture	3.4	6.8
	17	Distance from screw tip to medial cortex for distal screws (length of distal screws)	3.6	7.6
	18	Placement of proximal lag screw(s) across the intraarticular fracture	4.2	8.9
	19	Number of lag screws	3.4	8.0
	20	Trajectory of proximal screw(s) in the frontal plane (parallelity to the joint line)	3.7	8.2
	21	Orientation of proximal screw(s) in the axial plane	3.6	7.8
	22	Distance from screw tip(s) to medial cortex for the proximal screws (length of proximal screws)	3.9	8.5
End of procedure	23	Obtaining a correct lateral fluoroscopy view of the knee joint for documentation	4.4	8.8
	24	Obtaining a correct frontal fluoroscopy view of the knee joint for documentation	4.5	9.1
	25	Obtaining lateral fluoroscopy views with abduction and adduction to visualize the lateral and medial plateau	3.4	7.8
	26	Examination of the knee for ligamentous injury and range of motion after fixation	4.3	8.8

## Discussion

This Delphi process identified 26 assessment parameters from fracture preparation to procedural fluoroscopy documentation and ligamentous testing. These parameters were judged important by an international panel of experts and reflect both fundamental principles of osteosynthesis and clinical considerations specific to anterolateral buttress plate fixation. Across rounds, the initial 156 suggestions were consolidated to 31 parameters after content analysis, and five additional items were excluded in Round 2, yielding a final 26-item tool that represents stable consensus among experts.

Seven parameters pertain to fracture site preparation and fracture reduction. “Fracture site preparation” is essential for optimal fracture healing with minimal callus formation [14], and “medial percutaneous incision for reduction clamp placement” helps prevent unnecessary soft tissue damage, a factor linked to higher infection rates [15]. Haller et al. [16] highlight the importance of “direct visualisation of articular surface reduction” to minimise malreduction.

Four parameters relate to hardware choice, including “choice of plate length”, “choice of drill sizes”, “choice of screw type”, and “choice of screw diameter”. While the optimal screw type and size for buttress plate fixation in simple lateral TPFs is not well established, a combination of lag screws and buttress plating is recommended for managing meta-epiphyseal split fractures, as it helps protect the screws from shearing forces. Correct drill use is essential for optimal lag screw function [17].

Three parameters concerned plate placement and eight concerned screw placement. To achieve adequate buttressing and absolute stability, “contouring of the plate” is essential, as improper contouring may compromise interfragmentary compression and joint congruency [18]. Achieving an effective buttress effect also requires proper “placement of the plate relative to the direction of shearing forces”; specifically, the plate must span the apex of the fracture and maintain close contact with the bone [18]. Although not in a linear fashion, fixation rigidity is influenced by the “number of screws below the fracture”, which generally enhances stability but must be balanced against surgical exposure [19]. The appropriate “number of lag screws” depends on the specific fracture pattern and fragment length [20]. “Placement of proximal lag screw(s) across the intra-articular fracture” augments overall rigidity and compression across the intra-articular fragments. Additionally, the “orientation of proximal screw(s) in the axial plane” should be perpendicular to the fracture plane to maximise lag screw effect [21].

Four parameters pertained to the final steps of the procedure. Consensus was that correct lateral and frontal fluoroscopy views for documentation were important. In addition, “obtaining lateral fluoroscopy views with abduction and adduction to visualise the lateral and medial plateau” was considered necessary for sufficient postoperative documentation. Finally, given the risk of soft tissue damage with TPFs, “examination of the knee for ligamentous injury and range of motion after fixation” was identified as critical [22].

Although we believe the number of panellists completing each round was sufficient for a valid Delphi study, [11] both the recruitment and response rates were modest, which represent a limitation. The low recruitment rate may be attributed to sending survey invitations via email without prior inquiry. As a result, some recipients may not have met the inclusion criteria or have been unreachable. The deliberate inclusion of a large international panel may also have affected engagement, as participation often declines over time in Delphi studies requiring multiple rounds [11]. A further limitation is the limited variation in weight scores observed in Round 4. However, the strong and statistically significant correlation between Round 2 importance ratings and Round 4 weights (*r* = 0.92) indicates clear alignment between expert judgments of importance and weighting across rounds, supporting the internal consistency and content validity of the final parameter set.

A further limitation concerns the geographical distribution of panellists, which was uneven (Fig. 2), indicating that some regions were more strongly represented than others. As individual contact details and demographic information for non-responders were not accessible due to data-protection policies, the geographical composition of the invited sample was unknown, and geographical stratification was therefore not feasible. This imbalance may limit the broader generalizability of the findings, although the identified parameters reflect fundamental surgical principles that are likely applicable across many training environments. In addition, because only demographic information for respondents was available, any potential selection bias arising from the modest recruitment and declining response rates cannot be characterised, and the direction of such bias remains unknown.

To mitigate the risk of excluding relevant content, we employed less strict consensus thresholds than some comparable studies, recognising that overly strict cut-offs can eliminate essential parameters. This approach led to identifying 26 parameters. While this may reduce operational feasibility for clinical assessment, the primary objective was to develop a comprehensive tool that can later be refined or adapted for diverse educational environments. The identified parameters focus on bone management in anterolateral buttress plate fixation for a simple split fracture of the lateral tibial plateau without depression. Other aspects, such as soft tissue handling and closure techniques, were deliberately excluded, as these can already be reliably assessed using generic tools such as OSATS [23].

Although TPFs are less commonly handled by residents than other fractures during early surgical training, they require precise management and carry high stakes. When such procedures are performed infrequently, the quality of feedback becomes even more critical. Cognitive Evaluation Theory emphasises that intrinsic motivation is enhanced by experiences that promote a sense of competence, such as optimal challenge, supportive communication, and the absence of punitive evaluation [24]. Building on this, Wisniewski et al. synthesised data from over 435 studies and concluded that feedback is most effective when it provides high informational content, including insights into task execution, underlying processes, and self-regulation strategies [25]. The assessment tool developed in this study aims to support such feedback by offering structured, task-specific assessment parameters for simulated and clinical settings. It can thus guide supervisors in delivering targeted feedback, even during rare encounters. This aligns with broader goals in surgical education, where structured assessment and feedback are foundational to skill acquisition, trainee engagement, and implementation of CBME. Recognising the importance of structured feedback in clinical and SBT is imperative for the successful implementation of CBME in orthopaedic surgery. SBT offers a safe, structured environment in which residents can engage in deliberate, ongoing practice while receiving immediate, targeted feedback on their technical skills, without placing patients at risk [6].

By integrating assessment into both simulated and clinical settings, this tool supports skill refinement, performance evaluation, and individualised learning, contributing to safer, more effective training.

## Conclusion

This study provides a clearly defined assessment tool for evaluating technical competence in buttress plate fixation of tibial plateau fractures, developed through structured consensus from an international expert panel. The tool demonstrates strong content validity and can be applied in both simulated and clinical training to support deliberate practice and learner development. It also offers a foundation for high-quality, targeted feedback and may inform future virtual simulation platforms for automated performance analysis. With refinement and validation, it holds promise as both a formative and summative assessment resource that can ensure consistent, competency-based guidance throughout surgical training.

## Data Availability

No datasets were generated or analysed during the current study.
